# Missed for Years, Cured in Hours: Surgical Excision of a Popliteal Schwannoma With Intraoperative Neuromonitoring

**DOI:** 10.7759/cureus.94392

**Published:** 2025-10-12

**Authors:** Bharat R Dave, Mahesh Sagar, Mirant B Dave, Shivanand C Mayi, Hemant Saraiya

**Affiliations:** 1 Spine Surgery, Stavya Spine Hospital and Research Institute, Ahmedabad, IND; 2 Plastic and Reconstructive Surgery, Saraiya Plastic Surgery Hospital, Ahmedabad, IND

**Keywords:** intraoperative neuromonitoring, microsurgical excision, peripheral nerve sheath tumor, popliteal schwannoma, tibial nerve tumor

## Abstract

Popliteal schwannomas are rare benign tumors arising from the peripheral nerve sheaths, often misdiagnosed due to their nonspecific symptoms. We report a case of a 36-year-old female with a 10-year history of refractory left leg pain localized to the S1 dermatome, characterized by persistent burning and aching sensations along the plantar foot, heel, and posterior calf. Despite multiple consultations with various specialists over the years, her symptoms were repeatedly misattributed to lumbar spine pathology, resulting in delayed diagnosis. A detailed clinical evaluation eventually revealed a palpable mass in the popliteal fossa. MRI imaging showed a well-defined lesion consistent with a nerve sheath tumor arising from the tibial nerve. Histopathological examination after surgical excision confirmed the diagnosis of a tibial nerve schwannoma. Intraoperative neuromonitoring (IONM) was utilized to aid in the precise identification and preservation of nerve function during tumor excision. Neuromonitoring showed improved neural function with increased SSEP amplitude (e.g., from 0.41 µV to 0.72 µV) and decreased latency (e.g., from 47.9 ms to 43.4 ms) postoperatively in multiple channels, indicating enhanced nerve conduction. Additionally, transcranial motor evoked potentials (TcMEPs) were preserved after tumor excision, further supporting the functional integrity of motor pathways during surgery. The patient experienced complete symptomatic relief postoperatively, with no neurological deficits. This case underscores the importance of maintaining clinical suspicion for sinister pathology in patients presenting with long-standing, refractory leg pain and highlights the value of thorough clinical examination combined with appropriate imaging for the diagnosis and the importance of neuromonitoring during surgery.

## Introduction

Popliteal schwannomas are rare peripheral nerve sheath tumors, representing a very small minority of soft tissue neoplasms, with schwannomas in the lower extremities accounting for approximately 1% to 10% of cases. Within the popliteal fossa, schwannomas most commonly affect the posterior tibial nerve (around 8.9% of cases), while common peroneal nerve schwannomas are rarer and often smaller in size, contributing further to diagnostic challenges and delayed detection [1]. These tumors typically grow slowly and are well-encapsulated, usually appearing as single nodules [2]. Patients commonly experience symptoms such as localized pain, numbness, or swelling behind the knee. Because these symptoms are nonspecific, they are often mistaken for more common conditions like Baker’s cysts or lumbar spine problems. A helpful clinical clue is a positive Tinel’s sign, when light tapping over the affected nerve elicits tingling or shooting pain, indicating nerve involvement [3].

Unfortunately, diagnosis is often delayed. Many patients suffer symptoms for years, sometimes over a decade, and may see multiple healthcare providers before the correct diagnosis is made. This delay often arises because the tumor grows slowly and its symptoms can be subtle or mistaken for spinal issues. Imaging techniques such as ultrasound and especially magnetic resonance imaging (MRI) are crucial in detecting these lesions. On MRI, schwannomas usually appear as well-defined, encapsulated masses that follow the course of the affected nerve. However, the definitive diagnosis relies on histopathological examination, which reveals distinctive features: Antoni A areas, consisting of densely packed tumor cells; Antoni B areas, which are more loosely arranged and myxoid; and Verocay bodies, microscopic structures characteristic of schwannomas [4,5].

Tibial nerve schwannomas represent a small fraction, estimated between 1% and 10%, of all peripheral nerve sheath tumors [5]. Their rarity, combined with the diagnostic challenge they pose, highlights the importance of comprehensive clinical examination, including careful palpation and neurological assessment, and targeted imaging in patients with unexplained, persistent leg pain. These factors make reporting and studying such cases especially valuable.

This report aims to raise awareness of tibial nerve schwannomas, illustrate the diagnostic difficulties they present, and emphasize the critical roles of modern imaging and tissue diagnosis in guiding successful surgical treatment.

## Case presentation

A 36-year-old female software professional (BMI: 29) presented to the outpatient orthopedics clinic with a 10-year history of severe, progressive left leg pain that significantly affected her daily activities and caused sleep disturbances. Despite consultations with multiple specialists, including physiotherapists, neurophysicians, orthopedic surgeons, psychiatrists, and spine surgeons, her symptoms persisted. Previous investigations, including lumbar spine X-rays and MRIs, were unremarkable, and treatment with analgesics and neuropathic medications yielded only temporary relief.

Upon examination, the patient exhibited normal lumbar spine motion and lower limb power, with sensory blunting noted over the lateral three toes of the left foot. There was no visible swelling, but localized tenderness was observed over the left popliteal fossa, and painful terminal restriction of knee flexion was noted. Careful clinical palpation along the course of the sciatic nerve in the thigh and popliteal fossa revealed a tender, deep-seated mass eliciting sharp, radiating pain consistent with a positive Tinel’s sign. This clinical finding heightened suspicion of a peripheral nerve lesion. Distal pulses were palpable and equal bilaterally. Based on these findings and the chronicity of symptoms, further imaging was advised.

**Figure 1:**
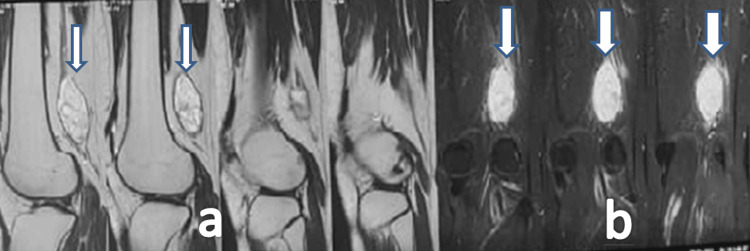
(a) The proton density image shows a well-defined, encapsulated isointense lesion with a hypointense capsule, as indicated by the arrows. (b) The STIR image demonstrates a hyperintense lesion, also indicated by the arrows. This well-defined, encapsulated lesion with a hypointense capsule is characteristic of a benign schwannoma, helping differentiate it from malignant or cystic masses commonly seen in other soft tissue tumors

An MRI of the left knee performed at 1.5T revealed a well-defined, encapsulated soft tissue mass (56 mm x 26 mm sagittal, 27 mm x 25 mm axial) in the posterior aspect of the knee, arising from the tibial division of the sciatic nerve. The lesion appeared well-defined and encapsulated on imaging, with the proton density sequence showing an isointense signal with a hypointense capsule, as shown in Figure 1a, while the STIR sequence demonstrated the lesion as hyperintense, as shown in Figure 1b. These features are consistent with a nerve sheath tumor, most likely a schwannoma [2].

Tinel’s sign was positive, eliciting radiating pain along the sciatic nerve distribution [6]. There were no bony abnormalities or meniscal pathology on radiographs. Based on the clinical and radiological correlation, a diagnosis of tibial nerve schwannoma was made. Differential diagnosis included other peripheral nerve sheath tumors, such as neurofibroma; however, the encapsulated nature, slow growth, and imaging characteristics supported a diagnosis of schwannoma.

**Figure 2:**
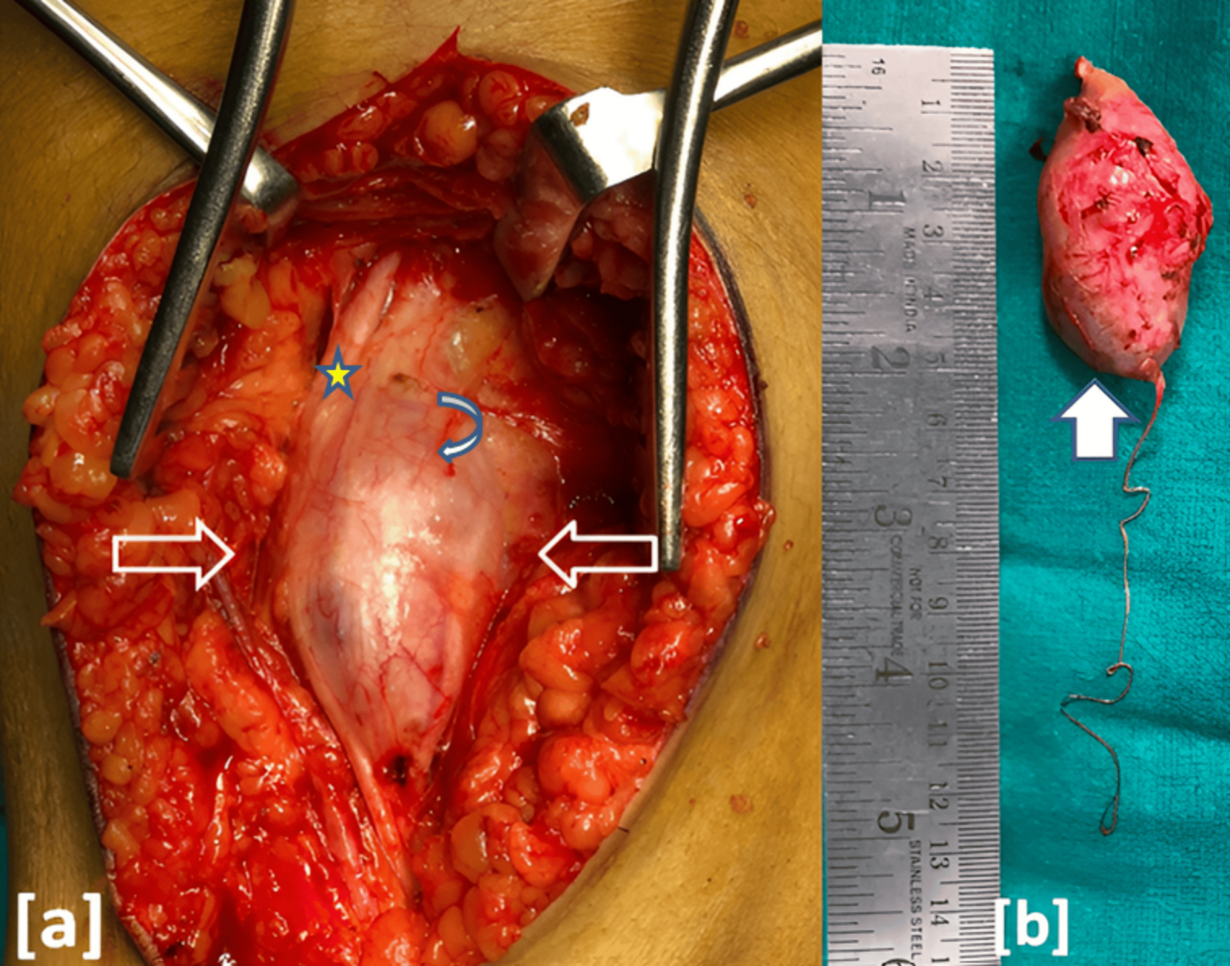
(a) Intraoperative view of the tumor mass in the left popliteal fossa, meticulously dissected and freed from surrounding tissues, as indicated by arrows. The tibial nerve is indicated by a five-pointed star, and the tumor capsule by a curved left arrow. (b) Excised globular tumor mass removed en bloc along with its parent nerve, as indicated by an arrow

Surgery was performed under general anesthesia (total intravenous anesthesia (TIVA)) with a tourniquet in the prone position, utilizing intraoperative neuromonitoring (IONM). TIVA offers advantages such as improved hemodynamic stability and faster recovery, which are beneficial in delicate surgeries requiring precise neuromonitoring and functional preservation. Additionally, its ability to reduce postoperative nausea and vomiting and allow rapid neurological assessment aligns well with the goals of peripheral nerve tumor excision to minimize patient morbidity and facilitate early postoperative evaluation. A midline incision was made over the popliteal fossa. The tumor was carefully dissected from the tibial nerve after identifying the proximal and distal ends, as shown in Figure 2a. Using microscopic and blunt dissection techniques, the nerve sheath was longitudinally incised to minimize fascicular injury, and the tumor was excised en bloc as shown in Figure 2b. Clear fascicular separation was observed. No intraoperative bleeding or complications were observed during the procedure.

**Figure 3:**
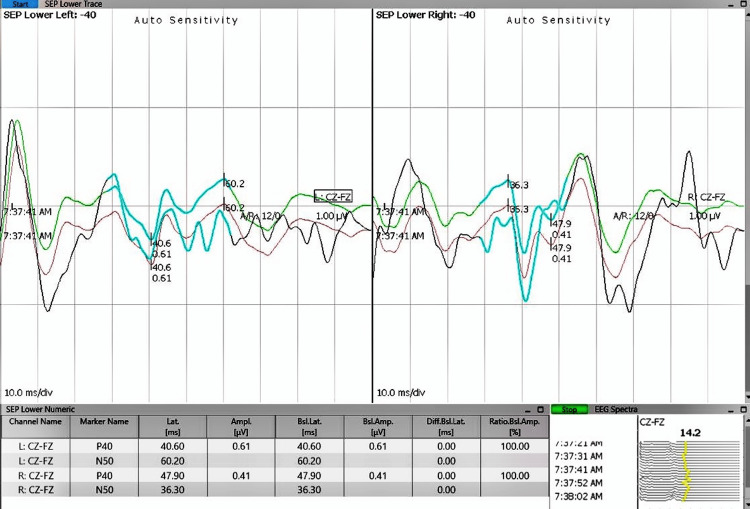
IONM recordings demonstrating SSEPs before surgical excision of the tumor mass. SSEPs measure electrical responses in sensory pathways SSEP: somatosensory evoked potential; IONM: intraoperative neuromonitoring

**Figure 4:**
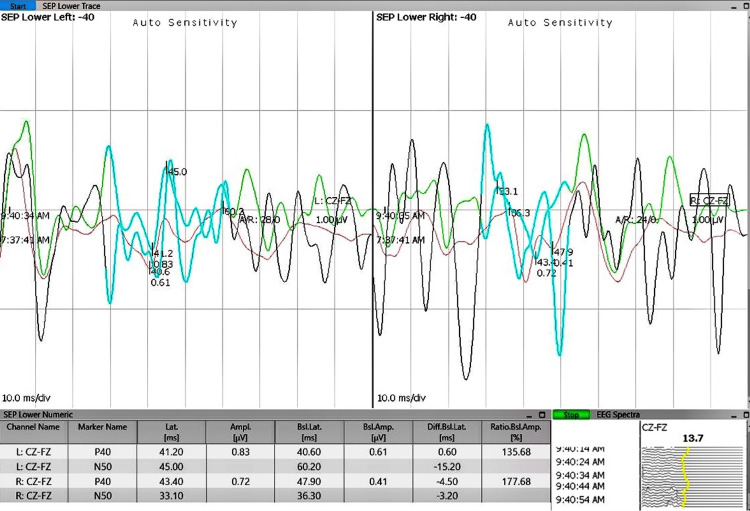
IONM recordings demonstrating SSEPs after surgical excision of the tumour mass, showing significant signal improvement post-excision SSEP: somatosensory evoked potential; IONM: intraoperative neuromonitoring

**Figure 5:**
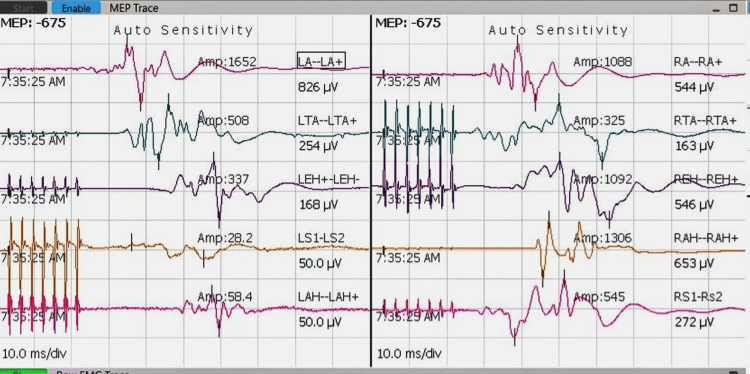
IONM recordings demonstrating TcMEPS before surgical excision of the tumour mass Preoperative TcMEP recordings provide a baseline assessment of the functional integrity of the motor pathways before surgical intervention, allowing for comparative intraoperative monitoring. Establishing this baseline is crucial for detecting changes during surgery that may indicate potential motor pathway compromise, enabling timely intervention to prevent permanent neurological deficits. TcMEPS: transcranial motor evoked potentials; IONM: intraoperative neuromonitoring

**Figure 6:**
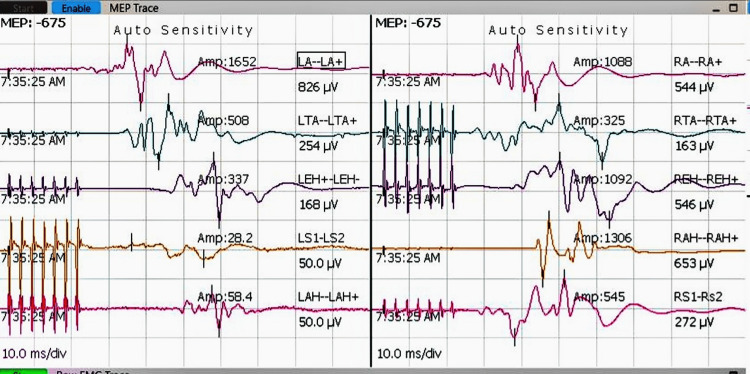
IONM recordings demonstrating TcMEPs after surgical excision of the tumor mass, showing preserved TcMEPs Monitoring TcMEPs is crucial for assessing the functional integrity of motor pathways during surgery, providing real-time feedback that helps prevent postoperative motor deficits. Preserved TcMEPs post-excision indicate maintained motor pathway function and suggest a successful surgical outcome with minimal risk of motor impairment. TcMEPs: transcranial motor evoked potentials; IONM: intraoperative neuromonitoring

Neuromonitoring demonstrated significant intraoperative changes in both somatosensory and motor pathways during tumor excision. As shown in Figure 3, somatosensory evoked potentials (SSEPs) prior to tumor removal represent baseline electrical responses in sensory pathways. Figure 4 depicts SSEPs after excision, revealing marked signal improvement, indicative of enhanced sensory pathway conduction. This improvement is supported by quantitative data on latency and amplitude presented in Table 1, where postoperative reductions in latency and increases in amplitude suggest preserved or improved nerve conduction and sensory function. Similarly, preoperative transcranial motor evoked potentials (TcMEPs), shown in Figure 5, establish a baseline for assessing motor pathway integrity before surgery, which is crucial for detecting intraoperative changes that might indicate motor compromise. Postoperative TcMEPs, as illustrated in Figure 6, demonstrate preserved motor pathway function, reflecting successful surgical management and minimal risk of motor deficits. Muscle groups monitored for TcMEP included tibialis anterior, extensor hallucis longus (EHL), S1 and S2 muscles of the perineal region, and abductor hallucis.

**Table 1:** Intraoperative SSEP data recorded using the Cz-Fz electrode montage for both left and right sides, comparing preoperative and postoperative latency (ms) and amplitude (µV) values Latency represents the time taken for sensory signals to travel from the stimulation site to the cerebral cortex, where a reduction postoperatively indicates improved conduction velocity. Amplitude reflects the magnitude of the cortical response, with higher values signifying better neural function. In the presented data, postoperative latencies generally decreased or remained stable, suggesting preservation or enhancement of nerve conduction after tumor excision. Simultaneously, amplitude increases postoperatively imply improved cortical responses and sensory pathway integrity. Overall, these changes suggest a functional improvement in somatosensory pathways resulting from surgical intervention and successful neural preservation. Cz-Fz channel means a bipolar recording between the central vertex (Cz) and frontal midline (Fz) electrodes. SSEP: somatosensory evoked potential

channel name	latency preop (ms)	latency postop (ms)	amplitude preop (µV)	amplitude postop (µV)
Left: CZ-FZ	40.6	41.2	0.61	0.83
Left: CZ-FZ	60.2	45		
Right: CZ-FZ	47.9	43.4	0.41	0.72
Right: CZ-FZ	36.3	33.1		

Intraoperative direct nerve stimulation using a bipolar probe aided in precisely differentiating functional nerve tissue from surrounding soft tissue. This technique facilitated the identification of nerve fascicles and guided safe tumor dissection, helping to minimize neural injury.

Together, these figures and data provide comprehensive evidence of functional improvement and neural preservation following tumor excision.

The patient experienced immediate relief of radicular leg pain. Mild numbness over the fourth and fifth toes was noted but improved within 48 hours. She was mobilized the next day and discharged in stable condition. At the three-year follow-up, she remained asymptomatic with no evidence of neurological deficits. This long-term outcome highlights the success of the surgical intervention and preservation of nerve function.

**Figure 7:**
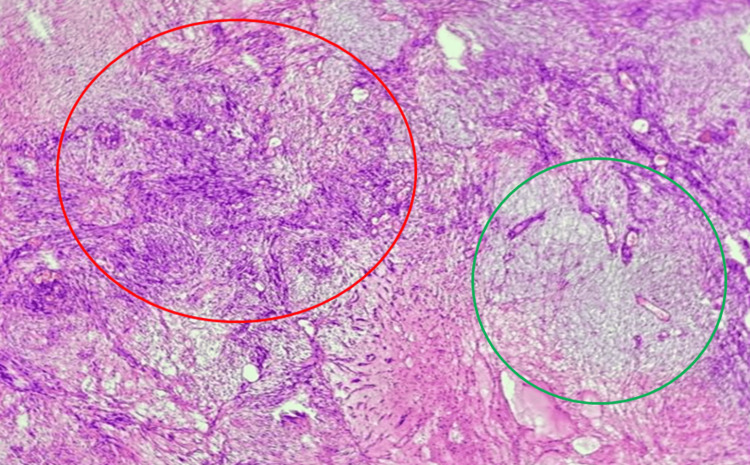
The image depicts a schwannoma exhibiting classic histological patterns The area within the red circle shows the Antoni A pattern, characterized by densely packed spindle-shaped Schwann cells arranged in compact fascicles with nuclear palisading. The green circle highlights the Antoni B pattern, which consists of loosely arranged Schwann cells within a myxoid and microcystic stroma, showing less cellularity and more extracellular matrix. These patterns are diagnostic features of schwannoma and reflect its biphasic tissue architecture.

Microscopic analysis revealed a well-encapsulated benign schwannoma with classic Antoni A and Antoni B patterns, as shown in Figure 7, as well as S100-positive immunohistochemistry, confirming the diagnosis.

## Discussion

Peripheral nerve tumors, although rare, should be considered in patients with chronic leg pain mimicking lumbar radiculopathy, especially when spinal imaging is normal and conservative treatments have failed [7]. Delayed diagnosis often results from incomplete clinical examination. A focused clinical examination plays a pivotal role in suspecting such lesions. This delay can prolong patient suffering and complicate timely treatment. In particular, localized tenderness, the presence of a palpable mass, and a positive Tinel’s sign along the nerve course should raise clinical suspicion. Palpation of the sciatic nerve along its course, especially at accessible points such as the gluteal region and the popliteal fossa, is crucial for detecting localized tenderness or mechanosensitization indicative of nerve inflammation. This examination complements other clinical tests by directly assessing nerve trunk sensitivity, helping to confirm nerve involvement in patients with leg pain when imaging is inconclusive. In our case, persistent symptoms and focal signs over the popliteal fossa prompted further imaging, which ultimately revealed a schwannoma of the tibial nerve. Schwannomas are typically slow-growing, well-encapsulated benign tumors arising from Schwann cells. They often remain asymptomatic until they compress adjacent nerve fascicles, producing neuropathic pain or paresthesia [2]. Peripheral nerve tumors can present with symptoms that closely resemble those of spinal pathologies, often creating diagnostic challenges and leading to potential delays in appropriate treatment [7]. Literature documents a delay up to several years before a correct diagnosis is made, underscoring the importance of thorough peripheral examination in all cases of radicular symptoms [8].

MRI remains the gold standard for diagnosis due to its ability to delineate soft tissue structures and accurately characterize them. Features such as the target sign, fusiform shape, and relation to the parent nerve help identify nerve sheath tumours [2]. While ultrasound can be a useful initial tool in the hands of experienced practitioners, it lacks the specificity to distinguish between schwannoma, neurofibroma, or malignant variants [2]. 

Sciatic MR neurography plays a critical role in the early diagnosis of small-sized peripheral nerve tumors by providing high-resolution images that delineate nerve anatomy and pathology with exceptional detail. Recent studies report sensitivity rates of approximately 94% and specificity around 92% for MR neurography in detecting peripheral nerve tumors, highlighting its high diagnostic accuracy compared to conventional imaging. This technique allows precise localization of lesions, assessment of nerve continuity, and differentiation from surrounding tissues, enabling timely surgical planning and intervention with improved outcomes [9].

Histological confirmation following excision remains essential, as imaging alone cannot definitively rule out malignancy. Histopathologically, schwannomas exhibit Antoni A and B patterns, nuclear palisading, and S100 protein positivity [10]. In our case, the tumor displayed classic features without atypia or necrosis. Surgical excision is the treatment of choice in symptomatic cases, especially when the mass causes pain, neurological deficit, or uncertainty in diagnosis.

IONM is crucial in the excision of popliteal schwannomas, providing continuous real-time feedback on the functional integrity of sensory and motor pathways. Monitoring SSEPs and TcMEPs aids in the early detection of neural compromise, allowing immediate surgical adjustments to prevent irreversible nerve injury. Improved postoperative SSEP latencies and amplitudes reflect enhanced sensory conduction, while preserved TcMEPs confirm maintained motor function, both correlating with favorable clinical outcomes. IONM has been shown to reduce postoperative neurological deficits in complex peripheral nerve surgeries by guiding precise dissection and minimizing trauma [11,12]. By integrating multimodal monitoring, surgeons can balance maximal tumor resection with optimal nerve preservation, significantly impacting patient quality of life [13]. In contrast, prior literature and surgeries without IONM report a higher risk of postoperative neurological complications, such as motor weakness or sensory deficits, owing to inadvertent injury to nerve fascicles during tumor resection [14].

Intraoperative direct nerve stimulation using a bipolar probe complements IONM by allowing precise distinction between functional nerve fascicles and surrounding soft tissues. This technique elicits localized muscle responses, facilitating mapping of nerve architecture and enabling tailored surgical dissection to avoid injury to critical fibers. By identifying functional nerve tissue intraoperatively, direct stimulation minimizes postoperative sensory and motor deficits and improves surgical outcomes [11,15]. Combining direct nerve stimulation with multimodal neurophysiological monitoring empowers surgeons with comprehensive functional guidance essential for safe and effective peripheral nerve tumor excision.

Postoperative outcomes are generally excellent, with minimal risk of recurrence or neurological complications when the tumor is completely excised [16]. In our patient, meticulous surgical technique led to complete symptom resolution and no functional impairment at follow-up.

This case highlights the importance of heightened awareness among clinicians and physical therapists regarding peripheral nerve tumors. Red flag signs, such as unexplained chronic neuropathic pain or focal deficits with routine spinal imaging, should prompt consideration of peripheral nerve pathologies. Early diagnosis and timely surgical intervention are crucial for optimal outcomes.

## Conclusions

Tibial nerve schwannomas, although rare, should be considered in patients with unexplained chronic leg pain despite normal spinal imaging. Careful clinical evaluation, including thorough palpation of the sciatic nerve and recognition of characteristic symptoms such as persistent radiculopathy and symptom chronicity, is essential to raise clinical suspicion. A focused clinical examination, supported by MRI and, when necessary, sciatic MR neurography, facilitates accurate diagnosis. Early surgical excision using microsurgical techniques usually results in excellent outcomes with minimal complications. The use of IONM further enhances surgical safety by providing real-time assessment of nerve function, enabling the surgeon to avoid neural injury and optimize functional preservation. Finally, histopathological analysis remains crucial for definitive diagnosis, guiding appropriate treatment and follow-up. This case highlights these points while acknowledging the inherent limitations of conclusions drawn from a single case.
